# Targeting hyaluronan accumulation in the tumor microenvironment

**DOI:** 10.18632/oncotarget.26483

**Published:** 2018-12-21

**Authors:** Xiaoming Li, Curtis B. Thompson

**Affiliations:** Halozyme Therapeutics Inc., San Diego, California, USA

**Keywords:** hyaluronan, hypoxia, tumor microenvironment, PEGPH20

It has been appreciated for some time that tumors are not simply clusters of malignant cells, but complex structures comprising cancer cells, non-malignant cells, such as cancer associated fibroblasts, endothelial cells and immune cells, and extracellular matrix (ECM) components, consisting of collagen, other matrix proteins and glycosaminoglycans. As a tumor grows, these malignant and non-malignant cells secrete growth factors, cytokines, and chemokines to promote the synthesis of an evolving ECM and induce the formation of vasculature. The tumor microenvironment (TME) that develops, is characterized by disorganized, leaky vasculature, poor lymphatic drainage and excessive ECM deposition, which favors cancer invasion and metastasis, attenuates anti-tumor immunity, and as first hypothesized by Rakesh Jain almost 40 years ago, acts as a barrier to drug delivery [1].

The predominant TME glycosaminoglycan, hyaluronan (HA), has been an area of focused research for over 30 years. HA is a non-sulfated, negatively charged, single chain megadalton glycosaminoglycan composed of repeating N-acetyl-D-glucosamine and D-glucuronic acid disaccharide units [2, 3]. Its simple molecular structure defines its physiochemical properties and unique biological role, namely its association with water to form a thick, viscous gel that resists compression and deformation [3]. Early clinical data demonstrated in several tumor types, including colorectal, lung, breast and pancreas cancer, that tumor HA accumulation is a negative prognostic indicator, with high levels of tumor HA being associated with shorter patient survival [3, 4]. This observation inspired early clinical trials that evaluated the degradation of tumor HA, using bovine hyaluronidase (BTH), in combination with existing therapies, across several solid tumor types [3, 4]. Although early data were promising, development of BTH was discontinued due in part to apparent allergic reactions to an enzyme of bovine origin [4].

**Figure 1:**
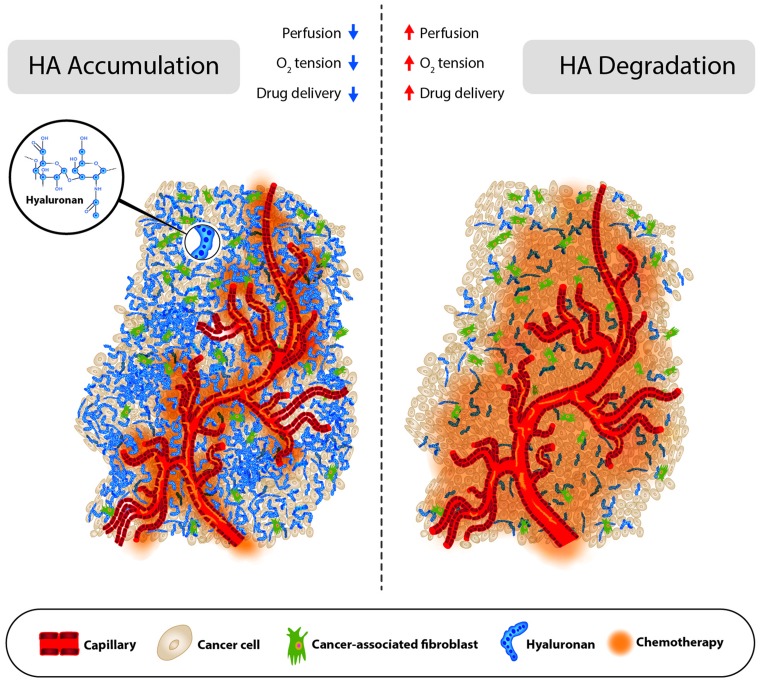
Pre- and post- hyaluronan degradation Schematic representation of a HA accumulating tumor treated with chemotherapy before (left) and after (right) PEGPH20 treatment.

HA has been linked to a variety of biological processes involved with tumor progression, including epithelial-mesenchymal transition, and the p53 tumor suppressor pathway, via its receptors, RHAMM and CD44 [2, 3]. Tumor HA accumulation also contributes significantly to elevated tumor pressures due to its gel-like properties, and indeed, preclinical models demonstrated that HA-accumulating tumors are characterized by high pressure, poor perfusion and poor drug accumulation [1, 5, 6]. Accordingly, therapies targeting tumor HA to reduce tumor pressure, increase perfusion and increase drug delivery have been ongoing since the early 1980s, when researchers first demonstrated that intratumoral BTH, by enzymatically degrading HA, could reduce tumor pressure, increase tumor perfusion and increase therapeutic delivery [4]. More contemporary studies using a systemic long-lasting hyaluronidase (PEGylated recombinant human hyaluronidase PH20; PEGPH20), demonstrated the same physiochemical changes (see Figure 1) [5, 6].

Recently, we extended earlier findings and further characterized the effects of HA accumulation and depletion on the TME [7]. To modulate tumor HA, we engineered tumor cells to overexpress HA synthase 3 (HAS3), one of three HA synthases (HAS1-3) responsible for synthesizing HA at the plasma membrane, which enabled us to compare HA ‘enriched’ tumors to their parental cell lines. We also treated tumor bearing mice with PEGPH20 and evaluated perfusion, hypoxia and drug accumulation. Increased tumor HA accumulation was associated with decreased tumor perfusion and increased tumor hypoxia. Post- PEGPH20, increases in tumor perfusion were pronounced and treatment significantly increased doxorubicin tumor penetration. We also observed a significant reduction in tumor hypoxia, which is linked with tumor aggressiveness via up-regulation of hypoxia-inducible factors, subsequent expression of pro-angiogenic proteins, and enhancement of the epithelial to mesenchymal transition (increased cellular migration). At high doses of PEGPH20, enzymatic HA degradation decreased HIF-1α protein expression, presumably due to a normalization in tissue oxygen concentrations from improved perfusion. Hypoxia has been linked to tumor associate macrophage (TAM) differentiation into T cell suppressive M2-like phenotypes [1], suggesting that reducing tumor hypoxia via HA degradation might mitigate T cell suppression.

As both HA and collagen contribute significantly to tumor pressure and both are associated with worsening prognosis, we also evaluated collagen levels contemporaneous with HA accumulation. In agreement with earlier observations [1, 8], tumor collagen I (Col I) increased concomitantly with tumor HA. We expanded our preclinical analysis to available human pancreatic cancer biopsies, where we observed a strong correlation between patient HA and Col I accumulation (*Pearson correlation coefficient* = 0.902), suggesting a role for both HA and Col I in the human disease. Elevated tumor pressure and the associated mechanical forces have been shown to stimulate collagen synthesis, likely through the signaling cytokine TGF-β, creating a tumor pressure positive feed-forward loop (i.e. higher pressure induces TGF-β, which induces collagen synthesis, which raises pressure, which induces TGF-β, etc.) [9]. The role of HA accumulation in this loop is unknown, but it likely also responds to mechanical compression, possibly through TGF-β, as HAS2 synthesis has been shown to be induced in mammary cells in response to TGF-β stimulation [10]. This may explain how losartan, an angiotensin receptor blocker, has been shown to reduce collagen and HA tumor accumulation, since it suppresses TGF-β levels [1]. A clinical trial is ongoing to evaluate losartan in patients with locally advanced pancreatic ductal carcinoma (PDA) [1].

Finally, since growth factors, cytokines and chemokines produced by malignant or non-malignant cells can concentrate in the gel-like ECM or bind to stromal components that have ionic charges, such as chondroitin sulfate and heparin sulfate proteoglycans, we recently began studies to understand how removing HA, and by default its water rich gel-like milieu, might impact cytokines instrumental to tumor progression. Whether as a result of reducing HA, or by disrupting binding to proteoglycans, we observed a complete wash out of recoverable VEGFA165 following PEGPH20 treatment [7]. Additional work is ongoing to characterize changes in TME signaling proteins, but this suggests another mechanism whereby HA degradation might mitigate the pro-tumorigenic TME.

Future studies will continue to increase our understanding of the role of HA accumulation in tumors and the TME changes associated with degrading tumor HA. At press, multiple clinical studies are ongoing to evaluate the therapeutic potential of degrading tumor HA, including a phase 3 clinical trial evaluating PEGPH20 with gemcitabine plus nab-paclitaxel in patients with metastatic PDA shown to accumulate HA (NCT02715804).
